# Neuroergonomic Stress Assessment with Two Different Methodologies, in a Manual Repetitive Task-Product Assembly

**DOI:** 10.1155/2021/5561153

**Published:** 2021-05-18

**Authors:** Ana García-Acosta, Jorge de la Riva-Rodríguez, Jaime Sánchez-Leal, Rosa María Reyes-Martínez

**Affiliations:** ^1^Tecnológico Nacional De México, Instituto Tecnológico de Ciudad Juárez, División de Estudios de Posgrado e Investigación, Av. Tecnológico No. 1340 C.P. 32500 Cd. Juárez, Chihuahua, Mexico; ^2^University of Texas at El Paso, Industrial, Manufacturing and Systems Engineering Department, 500 West University Ave, El Paso, TX 79968-00742, USA

## Abstract

Emotions are a fundamental part of mental health and human behavior. In the workplace, optimal performance of employees is necessary for productivity enhancements and its relation to the quality of a manufacturing product, therefore leading a company to advantages and competitiveness. This means that the workplace staff must remain in a neutral or a calm emotional state, for an adequate job performance. When an operation is not pleasant or the same task is carried out for a long period of time (repetitive), it can cause negative emotions such as stress, and this will have repercussions in poor work performance. The purpose of this research is, by means of an electroencephalogram (EEG), to identify the stress in the repetitive assembly of a manufacturing product. To measure brain waves, the Emotiv Epoc equipment was used and a manufacturing line was designed, divided into three workstations, where the assembly of product comprising a LEGO car was carried out within a manual repetitive approach. The appearance of stress was determined by employing two different methodologies, the prefrontal relative gamma marker (RG) and the valence, arousal, and dominance (VAD) emotional categories. The results obtained from the first methodology, corresponding to the RG marker, displayed a significant more change between the relaxation state and the product assembly carried out at 70% of the standard time (ST). A less significant change was observed between the relaxation state and the product assembly carried out at 100% ST, thus signaling the presence of stress. Additionally, the results from the VAD methodology resulted in moderate and low levels of stress, when the product assembly was carried out at 70% and 100% standard time, respectively.

## 1. Introduction

Stress is a disorder that continuously affects the population, regardless of age, marital status, gender, or activity. Our society needs people to be capable and trained to face and solve labor-related problems [1]. Overload work occurs when humans are subjected to more demanding activities outside of their ability to handle [2]. Nowadays, human well-being is considered a crucial aspect in every organization. Labor is considered an important wealth within every economic sector [3]. Therefore, stress identification represents an important research topic for psychologists and engineers [4].

Work-related stress is a problem that has increased in recent years; stress affects workers' health/increasing medical costs and also causes absenteeism problems, accidents, poor work performance, and manufacturing defects [5]. Many researchers have studied the phenomenon of stress over the years; one of the most accurate definitions is proposed by Lazarus and Folkman [6] as “a particular psychological relationship between the person and the environment that is evaluated by the person as something difficult or that exceeds their resources and endangers their well-being.” Stress can affect the organs and functions of the whole body (e.g., depression or anxiety, headache, and insomnia). There are different types of stress. First, acute stress occurs, which is the most common form of stress; symptoms may occur (e.g., irritability, depression, and tachycardia); however, when treated, the symptoms disappear easily. Next comes acute episodic stress, where the emotional state is irritable; it is always in a hurry and tends to be difficult. Finally, chronic stress is when stressors are present for several months or even years; illnesses can occur (e.g., gastritis, anxiety, insomnia, neurosis, and decreased performance) [7]. Not all stress is negative; there is positive stress that helps to perform tasks better called eustress. Eustress helps us to increase attention and performance capacity. When the activation exceeds the capacity of the human being, it becomes against the health of the person and lowers its performance [8].

Workers that continuously perform the same movements for a prolonged period of time often present problems in the neck, have muscle pain, and can feel tired and fatigue. Fatigue is named as a kind of distress due to the muscle exhaustion resulting from repetitive work [9]. The strain occurred by performing repetitive tasks for a long time produces injuries in joints, tendons, and muscles [5].

There are several approximate methods to measure stress, such as psychological questionnaires, physiological measures such as cortisol in the blood, and catecholamines. In the last 20 years, saliva testing has been the most popular method of measuring stress [7]. However, only some types of stress can be measured by techniques such as cortisol and heart rate.

Nowadays, stress research has focused on measuring neurological signals (EEG) that have a better temporal resolution, are noninvasive, economical, and rapid, and can be applied in real-time processing operations [10]. The EEG measures the electrical activity of the cortex of the brain to study its functionality. Through special electrodes, it records the electrical currents from the brain neurons [11]. The frequency characteristics are extracted from the neuronal activity of each person and are grouped into different bands (delta, theta, alpha, beta, and gamma) [12]. EEG studies have indicated that changes registered in the prefrontal regions and frontal areas through certain brain waves (e.g., Theta, Alpha, and gamma) are related to stress.

There exist two methodologies that have gained importance for detecting stress: the first one is based on a prefrontal relative gamma marker developed by Minguillon et al. [10] based on complementing fast and slow brain rhythms and the other methodology is based on emotional categories such as valence, arousal, and dominance, hereafter named “VAD” employed by many authors [13–15], where stress is categorized as a state of negative valence and positive arousal. Therefore, the level of arousal has an effect on personal performance, and the affective state of valence is associated with the level of stress. A high level of stress is related to positive arousal and negative valence, a moderate level appears with negative or positive arousal but with positive valence, and a low level occurred with negative valence and negative arousal [16].

Unfortunately, to the best of our knowledge, there are no well-established and reliable methodologies to detect stress in a manufacturing environment in real time, involving manual repetitive tasks in a product assembly [13]. The presence of repetitive and manual tasks throughout the work shift can cause musculoskeletal disorders (MSDs) of the upper extremities such as the forearm and wrist in workers and stress-related illness [17].

The main contribution of this work is the use of two markers in order to detect stress, the prefrontal relative gamma power (RG) [10] and the valence, arousal, and dominance (VAD) emotional categories [13]. These methodologies were applied and compared within a manufacturing line prototype for the assembly of a product composed of LEGO bricks [18], involving manual repetitive tasks, to mimic a real manufacturing environment. The results from this work could be helpful for the implementation of neuroergonomic strategies to improve work performance, diminishing musculoskeletal disorders as well as reducing adverse emotional conditions in the manufacturing industries.

The remaining sections of this work are organized as follows: the literature review is presented in Section 2, the experimental methodology is described in Section 3, the results derived from the statistical analysis using both the prefrontal RG marker and VAD marker are displayed in Section 4, the discussion of the significant results is presented in Section 5, and finally the summarized conclusions from this work are given in Section 6.

## 2. Literature Review

Recently, the electroencephalogram has become an easy-to-use and affordable tool; its use consists of three steps: (1) emotion capture, where people are presented with stimuli while EEG signals are recorded; (2) data processing, where signals are recorded under noise cancellation; (3) classification, where the relevant characteristics are extracted according to the classification method [19].

**Figure 1:**
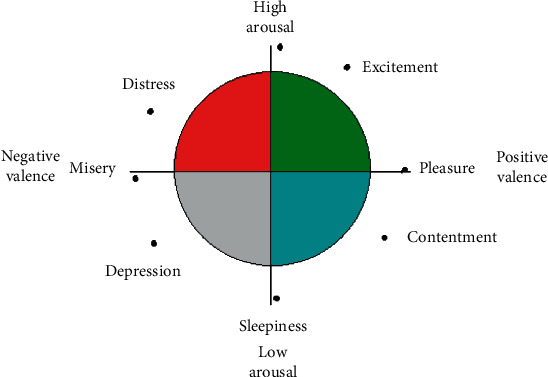
Emotional categories [21].

Through the brain-computer EEG interface, a wave analysis can be performed, and classification of the emotions can be carried out as well as stress level detection. Brain signals are relatively weak in the range of 0 to 100 Hz. One of the advantages of analyzing stress using EEG is that it is a noninvasive technique [20]. The cognitive representation of the emotions has eight variables, represented within a circle, in a two-dimensional space, as shown in Figure 1. In this spatial figure, the horizontal dimension, pleasure, or enjoyment are localized in the eastern part and disgust or anger in the west region. In the vertical dimension in the northern part, the arousal (agitation or excitement) is located, and in the southern part, the state of drowsiness is located [21].

According to recent investigations, the study of emotions with brain-computer interfaces has received considerable interest. To detect emotions regarding arousal and valence, the activity of alpha and beta waves was analyzed, by measuring EEG signals in the prefrontal cortex, corresponding to the AF3, AF4, F3, and F4 electrodes [13].

Alpha waves are more dominant in a relaxed state, while beta waves are associated with a state of agitation or alertness [14]. Also, alpha waves predominate during a state of mental relaxation and are most visible over the occipital and parietal lobes. The intense activity of the alpha waves has been correlated with the inactivation of the brain. In other words, the activity of the beta wave is related to the active state of the mind; it is more predominant in the frontal cortex during intense mental activity. For this reason, the beta/alpha ratio is an indicator of people's arousal status. To define the level of the valence, if the state of mind is negative or positive, the activation levels of the two cortical hemispheres are compared [13].

Valence is the regulation of emotion and conscious experience; the prefrontal lobe has a crucial role (F3 and F4). The inactivation of the right front side indicates a positive emotion, just as the inactivation of the left front side indicates a negative emotion [14]. The frontal inactivation of the left side is an indicator of a withdrawal response, associated with negative emotion. Also, the frontal inactivation of the right hemisphere is associated with an approximation response and related to positive emotions [13]. The domain is characterized by an increase in beta/alpha radius in the frontal lobe and an increase in beta waves in the parietal lobe [14].

Alpha and beta wave activities can be used to detect emotions (arousal and valence). Ramírez and Vamvakousis in their article indicated that Choppin [22], in 2000, proposed the use of EEG to classify six emotions employing neural networks. Choppin's approach is based on classifying emotions through valence, arousal, and a domain of the EEG signals. It characterized positive emotions by a high coherence in the alpha wave in the frontal region and high power of the beta wave in the right parietal region. High arousal was characterized by the highest potency of the beta wave and coherence in the parietal lobe, plus the lowest activity of the alpha wave, while the dominance (the strength of emotion) is characterized by an increase in the radius of beta/alpha activity in the frontal lobe, plus an increase in beta activity in the parietal lobe [13]. In the literature review, it was found that there are different ranges to determine emotions using the VAD methodology. According to different authors, Hosseini and Khalilzadeh [23] measured emotions using the “International Affective Picture System IAPS,” and to determine the level of arousal and valence, they used a scale from 1 to 9 based on the following intervals: calm state represented with arousal values <4, and valence in a specific range (4 <valence <6), negative excitations correspond to arousal values >5, and valence values <3. Instead, Verma and Tiwary [24] divided VAD into three categories using a continuous range of 1–9: low valence, arousal, and dominance (1–4.5); medium valence, arousal, and dominance (4.5–5.5); and high valence, arousal, and dominance (5.5–9), as shown in Table 1.

**Figure 2:**
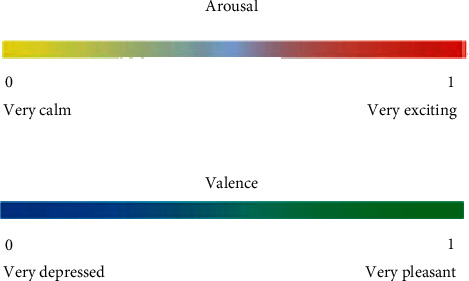
Range of valence and arousal values associated with emotional states [25].

Cui et al. [25] used the range between 0 and 1 in their studies to measure emotions based on valence and arousal. In arousal, there is a continuity from the calmest (0) to very exciting (1); the same continuity is present in the valence with the value of zero (0) corresponding to the most depressed state and a value (1) corresponding to the most pleasant state, as shown in Figure 2.

One study, related to manufacturing using EEG, was found in the literature, with regard to a study conducted in Serbia in 2015 using EEG in conjunction with the wireless SMARTING equipment (mBrain Train). The experiment consisted of studying whether workers' attention can be enhanced, by instructing them with which hand to start the assembly operation instead of choosing any hand freely [26]. The results of this experiment showed that the attention level was increased with the help of instructions.

## 3. Methodology

### 3.1. Subjects

The experiment in this work was carried out with 6 volunteer students, between 19 and 25 years old (5 men and one woman). None of the participants had neurological disorders, and they were alcohol, drugs, and medications free. All participants signed the informed consent, expressing their voluntary participation in the experiment and their disposition to leave at any time.

### 3.2. Experimental Measurements

In this research, a simulation of a manufacturing line was carried out by assembling a car with LEGO's pieces in an ergonomic workstation, mimicking the real conditions of a work area and recording brain waves to determine stress. LEGO is a system of building bricks that can be freely joined between two parts of LEGO and remain together until being separated and has been used in the simulation of manufacturing systems to teach Lean Philosophy [27]. Also, LEGO bricks have been employed for educational purposes in the design and management of manufacturing systems focused on an integrated approach [28].

### 3.3. Procedure

**Figure 3:**
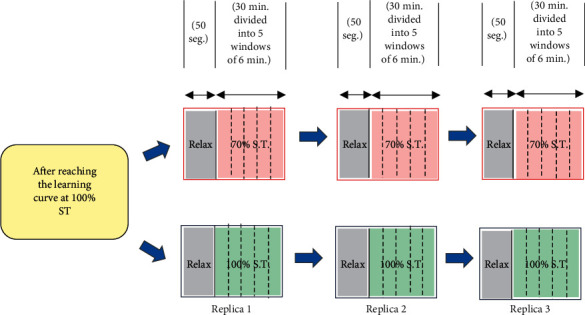
Experimental methodology for each participant.

The manufacturing line was divided into three stations, and the experiment was carried out using two standard times, one of them at 100% and the second at 70% percentage (this last time was used as a stimulus to cause stress in the assembly of a LEGO-based product). The ST corresponds to 2 minutes and 28 seconds (100% ST). The operational time (ST) was decreased from 100 to 70% (30% less); this means that each person needed to assembly the product more rapidly. The experiment was planned with 6 random people, 2 standard times, and 3 replicates, as shown in Figure 3, where EEG signals were recorded for the relaxation and assembly time. It is important to emphasize that each EEG epoch of 30-minute length was subdivided into five windows, therefore, obtaining a total of 15 EEG data per person, resulting in 90 brainwave values in total for each product assembly time (in total = 90).

The experiment started at 10 : 00 a.m. and ended at 2 : 00 p.m for a period of several weeks. The Emotiv Epoc equipment [29] and the Emotiv Pro Software were used for the recording of brain waves [30]. It was ensured that during the EEG recording there was good contact and good signals for all the electrodes.

At the beginning of the experiment, the people were trained to get acquainted with the Emotiv Epoc and to minimize artifacts that can occur by body movements (e.g., talking, smiling, and/or any unnecessary movement) while recording in the EEG experiment.

**Figure 4:**
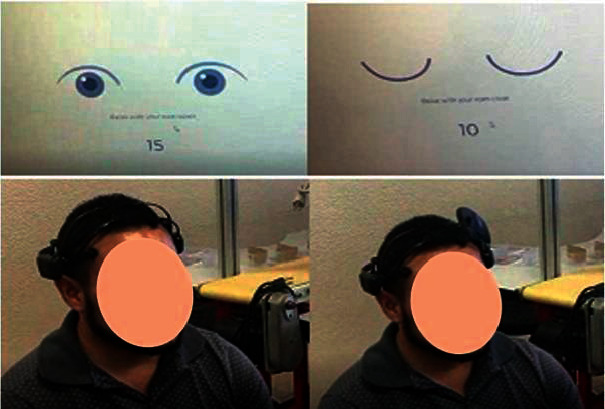
EEG recording during the relaxation state.

Before carrying the assembly task, each person was at rest, and in order to eliminate artifacts related to eye movement, 15 seconds were recorded with eyes open and 15 seconds with eyes closed, as shown in Figure 4, and the brainwaves were saved using the Emotiv Pro software. Furthermore, another 20 seconds was added to the relaxation time before starting the assembly operation, resulting in a total time of 50 seconds.

**Figure 5:**
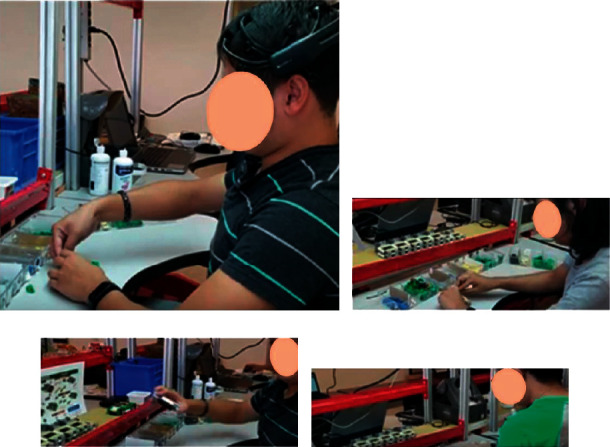
Assembly of a LEGO-based product and brainwaves recording.

Given that the assembly activities for the participants are very similar within each of the workstations, the product assembly was performed only in workstation 2, involving a time length of 30 minutes. The assembly operations consisted of “picking up the LEGO and assembling it.” A difficulty encountered during the operations arises because the LEGO pieces that are handled are small and also all the movements involving the assembly process are repetitive, as shown in Figure 5.

### 3.4. Analysis of EEG Signals

#### 3.4.1. Preprocessing

For the recording of the EEG signals, the Emotiv Epoc device was used, containing 14 channels (electrodes), spaced at the scalp in accordance with the international 10–20 system. The channels were labeled as follows: AF3, F7, F3, FC5, T7, P7, 01, 02, P8, T8, FC6, F4, F8, and AF4. It has two reference electrodes CMS/DRL, noise cancellation settings at P3/P4 locations, a sampling rate of 128 Hz, a bandwidth of 0.16–43 Hz, and digital notch filters at 50 Hz and 60 Hz. The resolution is 16 bits (14 bits effective) [29]. The two references CMS and DRL electrodes must be placed behind the ear, close to or above the mastoids; this is essential for its correct functionality and to assure adequate electrical signals [11].

The electrical activity produced by the brain is measured in units of microvolt; also, due to power line interferences and external interferences, the recorded EEG signals usually contain noises, produced by artifacts involving the eye (Electrooculogram), muscle (Electromyogram), and vascular movements (Electrocardiogram). Consequently, it is necessary for the preprocessing of the signal to remove these noises [31]. All processing and data analysis was performed with the MATLAB software environment [32]. The EEG data were preprocessed with EEGLAB 14.1.1 toolbox [33].

First, the data components were removed using independent components analysis (ICA). Second, the artifacts correction was performed using the Artifact Subspace Reconstruction (ASR) method [34]. ASR rebuilds the missing data with a spatial mixing matrix and uses an algorithm to remove nonstationary high variance in EEG signals. The ASR criteria for identifying a bad channel and its removal were the following: (a) the presence of flat line length of more than 5 seconds, (b) high-pass filter transition bandwidth (0.25–0.75 Hz), as signals at frequencies below 0.25 were removed, (c) if a channel has a correlation of less than 80% with respect to its activity based on the other channels, and (d) if a channel has more line noise relative to its signal in excess of four standard deviations relative to the overall channel population [35]. The EEG signal has a dissimilar frequency at different time intervals because the signals are nonstationary [36].

The domain frequency analysis was performed using the Fast Fourier Transform (FFT) algorithm to calculate the power spectral density (PSD) with units of *μV*^2^/Hz at various frequency intervals (Hz) for the EEG data and plotted using the EEGLAB graphical interface. The brainwaves are classified according to their frequency as follows: delta (1–4 Hz), theta (4–8 Hz), alpha (8–13 Hz), beta (13–30 Hz), and gamma (30–50 Hz) [37]. An in-house MATLAB script was developed to extract the PSD values in tabular form at different frequencies. The spectral analysis of signals, including removal of both noise and outliers, has also been applied, in other scientific areas such as seismology for the prediction of earthquakes, tsunamis, and volcano eruptions; speech recognition; radar and sonar systems; and control systems, to characterize the dynamical behavior of a given system, and for monitoring the wear of different mechanical parts [38]. For example, in the area of mechanics, Castaño et al. [39] employed a sensor designed to monitor the machining and milling operations on conductive materials. Beruvides et al. [40] applied the spectral analysis of signals for the detection of run-out in the microdrilling processing of metals.

Also in this work, in order to compare the brainwaves behavior within the different periods of time, where the assembly was carried out, each EEG recording length of 30 min was divided into 5 windows of 6 minutes each. The RG and VAD analyses were performed in the prefrontal area (AF3, F3, F4, and AF4) since this area has been used in previous studies to reveal emotions using the valence and arousal methodology [13–15]. Channels P8, FC6, and F8 were used for the dominance analysis [14, 19], and the values were averaged across these channels and analyzed together. The RG was calculated by taking the ratio of the gamma power (30–50 Hz) with respect to the power average of low rhythms (4–13 Hz). The spectral analysis using the RG is based on the work of Minguillon et al. (2016) [10] who elucidated it as an efficient and robust marker to measure stress, based on the VAD ranges obtained by two researchers [24, 25] and shown in Table 2.

In this work, the data were normalized by using the maximum and minimum values and the following formula *y*_norm_=([*y* − min(*y*)]/[max(*y*) − min(*y*)]) [10], where *y* is the entire feature set, min(*y*) is the minimum value in the feature set, and max(*y*) is the maximum value in the feature set, respectively [36]. The data from each person were normalized to reduce interparticipant variability (differences between participants) on a scale between 0 and 1 [41].

### 3.5. Data Analysis

All statistical analysis was performed with the Minitab Software [42]. First, the RG was calculated for the AF3, AF4, F3, and F4 channels for each of the five EEG windows, each window having a 6 min time length, at different ST and relaxation states for every person. Subsequently, the outliers were eliminated from the data according to three standard deviations criteria.

The Anderson Darling normality test was used to evaluate whether the data conforms to a normal distribution, giving rise to *p* values <0.05. Then, a Student's *t*-test was applied with a significance level *α* = 0.05 to determine differences between 70% ST versus relaxation and 100% ST versus relaxation states. Finally, the mean power of the RG of all people was calculated and normalized within the corresponding time period for all cognitive states: relaxation, 70% ST, and 100% ST, and the Student's *t*-test was applied to determine the statistically significant differences between the different states.

For the VAD methodology, an analysis of variance (ANOVA)-General Linear Model was performed applying the following factors: people and ST (relax, 70%, and 100%). Subsequent responses (normalized) were valence, arousal low beta and high beta, and dominance. The outliers were removed from the data. All results had *p* value <0.05 as shown in Table 3, indicating the existence of a statistically significant effect of each response variable (VAD) for the factors (people and ST) with *p* < 0.05.

## 4. Analysis of Statistical Results

The statistical analysis results for the prefrontal RG marker corresponding to each person are presented in Table 4.

**Figure 6:**
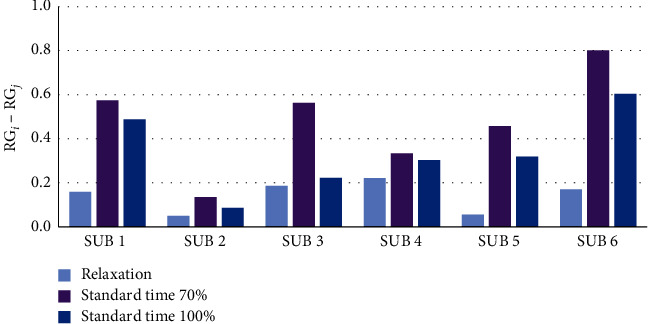
Prefrontal mean RG values during relaxation states and product assembly at 70% (subindex *i*) and 100% (subindex *j*) standard times.

The graphical representation of the average normalized prefrontal RG values for each state is shown in Figure 6, where it can be observed that subjects 1 and 6 have higher RG values in comparison with other persons at 70 and 100% ST.

**Figure 7:**
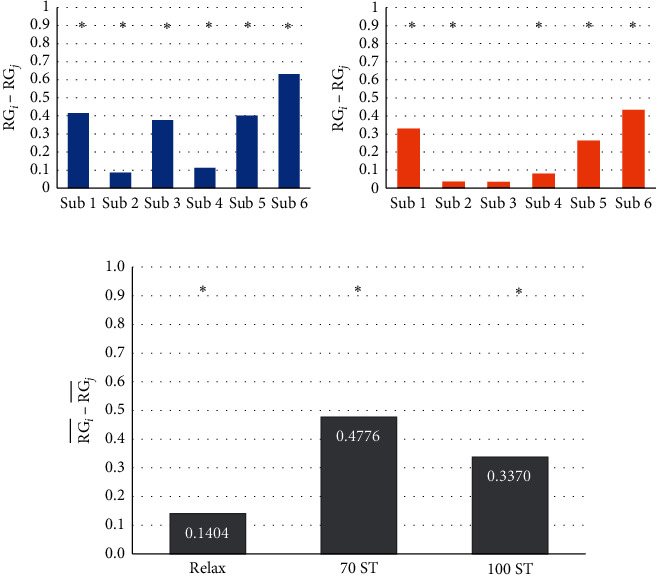
(a) The top left figure displays the RG differences between relaxation and product assembly at 70% ST, where the *i* subindex corresponds to 70% and *j* corresponds to relaxation. (b) The top right figure displays the RG difference between relaxation and product assembly at 100% ST, where *i* corresponds to 100% ST and *j* corresponds to relaxation. (c) The figure at the bottom shows the RG subject-averaged differences between product assembly at 70 and 100% ST against the relaxation state, where *i* and *j* correspond to relaxation, 70% ST, and 100% ST.

Figure 7(a) displays the calculated difference in the RG prefrontal mean values between 70% ST and relaxation for all people. The difference was statistically significant (Student's *t*-test; *p* < 0.05). Person 6 presented the highest difference, followed numerically by persons 1, 5, and 3. On the contrary, persons 2 and 4 displayed the smallest differences.  Figure 7(b) shows the calculated difference in the RG prefrontal mean values between 100% ST and relaxation; regarding persons 1, 2, 4, 5, and 6, the difference was statistically significant, except for person 3.

Figure 7(c) shows the normalized RG value averaged for all six persons; in the left part, the state of relaxation presents a lower RG value with respect to 70 and 100% ST. The highest RG value was at 70% ST. The calculated RG average difference was statistically significant between the relaxation state and the product assembly at 70 and 100% ST.

Table 5 presents the numerical *p*-values corresponding to the RG differences for each person between the relaxation state and 70% ST; all the differences are statistically significant (Student's *t*-test; *p* < 0.05). With respect to the calculated RG difference between relaxation state and 100% ST, it can be observed that for persons 1, 2, 4, 5, and 6 the differences were statistically significant, with the exception of person 3, where the difference was not statistically significant.

**Figure 8:**
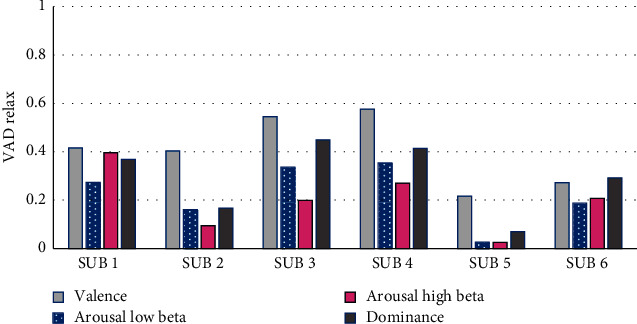
Valence, arousal, and dominance values during the relaxation state.

The valence, arousal, and dominance (VAD) methodology proposed by Blaiech et al. [14] was also used in this work in order to detect stress. These three factors or categories classify the seven states of emotions as follows: disgusted, enjoyed, surprised, sad, scared, angry, and neutral. To determine the valence and arousal values, the EEG data from electrodes AF3, AF4, F3, and F4 was employed, and for the domain, the information from electrodes P8, FC6, and F8 was used. In Figure 8, the VAD values for every person in the relaxation state are observed, where the values of low arousal and high beta for all people were <0.45, high valence (HV) values >0.45 were present for persons 1 to 4, and persons 5 and 6 displayed low valence (LV) <0.45, signaling a lower stress level.

**Figure 9:**
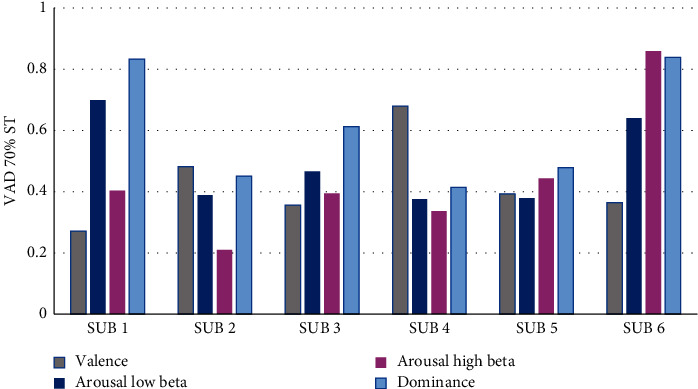
Valence, arousal, and dominance values during the product assembly at 70% ST.

Figure 9 displays the VAD values corresponding to the product assembly using a standard time of 70%. From this figure, it can be observed that persons 1 and 6 presented large arousal low beta (HALB) values >0.55, and only person 6 presented large arousal high beta (HAHB) values. Person 3 presented a medium arousal low beta value (MALB) of 0.46, and persons 2, 4, and 5 had values <0.55, which correspond to the low arousal low beta (LALB) regime. Persons 1, 2, 3, 4, and 5 had low arousal high beta (LAHB) values <0.45. Persons 1, 3, 5, and 6 presented low valence (LV) values, person 2 presented a median valence (MV) value, and finally, person 4 presented a high valence (HV) value. The analysis of these VAD results for the product assembly at 70% ST showed that persons 1 and 6 presented a high stress level with arousal low beta (ALB) values. The analysis corresponding to arousal high beta (AHB) values showed that only person 6 presented a high stress level.

**Figure 10:**
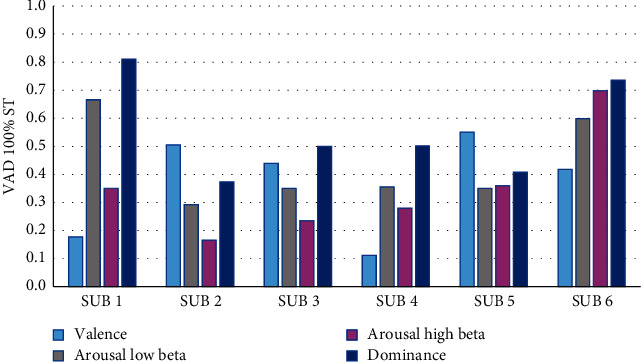
Valence, arousal, and dominance values during product assembly at 100% ST.

Figure 10 displays the behavior of the VAD values with respect to the product assembly at 100% ST. Persons 1 and 6 had HALB values >0.55, and the other people got a LALB <0.45. Only person 6 got HAHB, and the other persons obtained LAHB values. Person 2 presented a medium valence (MV) value, person 5 presented an HV > 0.55, and also persons 1, 3, 4, and 6 got an LV. The summarized results of the analysis for the product assembly at 100% ST indicated that persons 1 and 6 showed a high stress level with arousal low beta (ALB) values; with respect to the arousal high beta (AHB) values, only person 6 showed a high stress level, since this person displayed a faster assembly's ability compared to the other persons.

## 5. Discussions

The RG marker has been used previously to evaluate meditation states and stress [10]. The VAD categories had also been used to determine emotions (stress) [14]. In the experiment carried out in this work, two methodologies RG and VAD were used to detect stress in the assembly of a product involving manually repetitive tasks.

**Figure 11:**
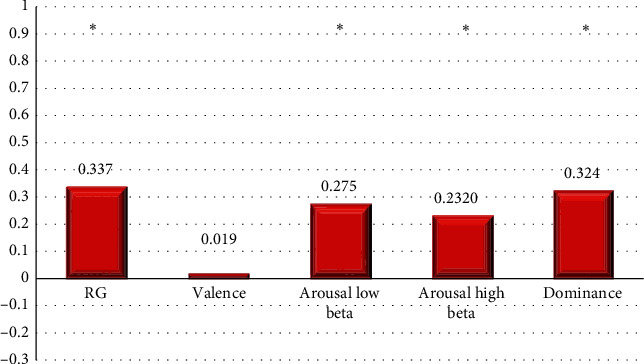
Differences in RG and VAD markers' mean values between relaxation and product assembly at 70% ST.

The results of the VAD methodology showed the presence of a high level of stress with respect to persons 1 and 6 in the assembly of 70% ST using ALB. Consequently, in the assembly of 100% ST, only person 6 presented a high stress level.  Figure 11 shows the difference between the mean power of the RG and VAD markers, for all six subjects at 70% ST versus relaxation, where most of the markers display a significant statistical difference, with the exception of the valence category.

**Figure 12:**
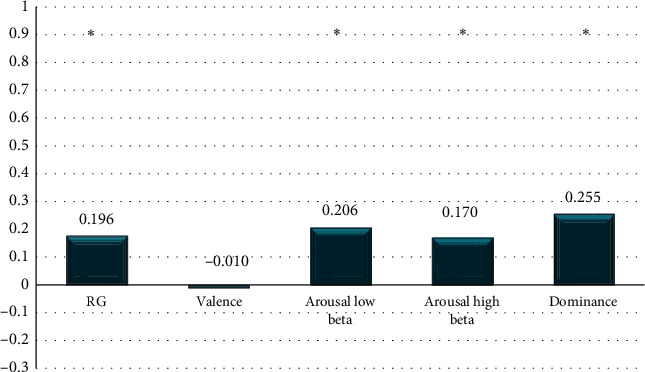
Differences in RG and VAD markers' mean values between relaxation and product assembly at 100% ST.

In Figure 12, the difference of the mean powers for the RG and VAD markers for all six persons at 100% ST versus relaxation is shown. It can be observed that the difference was not statistically significant for the valence category. The numerical differences in this figure (100% ST) are smaller in comparison to the values displayed in Figure 11 (70% ST).

**Figure 13:**
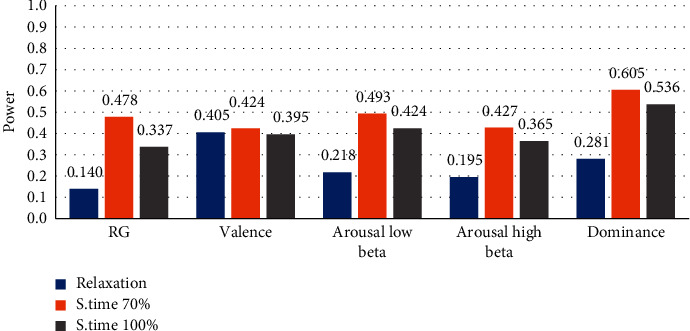
Summary of all average marker differences between relaxation and product assembly at 70 and 100% ST.

Finally, Figure 13 summarized the subject-averaged brainwaves power, with the RG and VAD methodologies, for the product assembly using manually repetitive tasks at different operational conditions and relaxation.

## 6. Conclusions

RG is a marker, based on complementing fast and slow brain rhythms, and has been used to detect stress [10]. Comparing the two methodologies (VAD and RG) in this work, it can be concluded that it was possible to detect the presence of stress. Also, it was noticed that the RG marker showed significant changes in the relaxation state versus 70 and 100% ST, respectively.

The results regarding the VAD methodology were the following: the values of the valence were similar within the three states (relaxation, 70%, and 100% ST). The ALB and AHB at 70% ST are higher than ALB and AHB at 100% ST. The beta brainwave presented more activity when people carried out the assembly at 70% ST, since the beta/alpha ratio is an indicator of arousal state [15]. The medium arousal range was 0.45–0.55; in the assembly, at 70%, we obtained a mean for ALB = 0.493 and AHB = 0.427, mean valence = 0.424, and mean dominance = 0.604. Therefore, it can be concluded that within 70% ST, the arousal low beta and high beta showed that people were working at optimal performance, therefore, signaling a moderate level of stress (low valence, medium arousal, and high dominance). In the assembly at 100% ST, a mean value in ALB = 0.424 and AHB = 0.365, mean valence = 0.395, and mean dominance = 0.536, the arousal low beta and high beta were obtained, indicating that persons were working at a low level of stress (low valence, low arousal, and medium dominance). In the relaxation state, the following values were obtained: the mean in ALB = 0.218 and AHB = 0.195, mean valence = 0.405, and mean dominance = 0.281. It can be concluded that in a relaxed state according to the arousal low beta and high beta, persons were in a very insignificantly low level of stress state according to Giannakakis et al. [16], and there were LV, LA, and LD categories.

In summary, during the assembly of a product with LEGOs, while performing manually repetitive tasks, the persons in the experiment presented a moderate level of stress within the 70% ST, since they were in an optimal performance state (LV, MA, and HD). On the other hand, people who worked at 100% ST presented low levels of stress level (LV, LA, and MD); with respect to the relaxation state, the persons presented insignificantly low levels of stress (LV, LA, and LD).

The results in this work indicated that both RG and VAD markers displayed similar behavior in the detection of stress and provided reference marker values, hence, signifying that when the persons were working at 70% ST, a higher level of stress was detected in comparison to those working at 100% ST.

These results could be helpful in the design of neuroergonomic strategies to improve work performance, diminishing musculoskeletal disorders as well as reducing adverse emotional conditions in the manufacturing industries involving manually repetitive tasks.

## Figures and Tables

**Table 1:** Valence, arousal, and dominance categories for emotions [24].

Valence class (range)	Arousal class (range)	Dominance class (range)
Low valence (1–4.5)	Low arousal (1–4.5)	Low dominance (1–4.5)
Medium valence (4.5–5.5)	Medium arousal (4.5–5.5)	Medium dominance (4.5–5.5)
High valence (5.5–9)	High arousal (5.5–9)	High dominance (5.5–9)

**Table 2:** Normalized values for the valencia, arousal, and dominance emotional categories in this study.

Valence (range)	Arousal (range)	Dominance (range)
Low valence (0–.45)	Low arousal (0–.45)	Low dominance (0–.45)
Medium valence (.45–.55)	Medium arousal (.45–.55)	Medium dominance (.45–.55)
High valence (.55–1)	High arousal (.55–1)	High dominance (.55–1)

**Table 3:** Summary of experimental ANOVA *p* values.

VAD				
	Valence	Arousal low beta	Arousal high beta	Dominance
Person	*p* ≤ 0.001	*p* ≤ 0.001	*p* ≤ 0.001	*p* ≤ 0.001
Standard time	0.002	*p* ≤ 0.001	*p* ≤ 0.001	*p* ≤ 0.001
Person^∗^ST	*p* ≤ 0.001	*p* ≤ 0.001	*p* ≤ 0.001	*p* ≤ 0.001

**Table 4:** Prefrontal mean values of the RG marker during relaxation states and product assembly at 70 and 100% standard times.

Mean RG	SUB 1	SUB 2	SUB 3	SUB 4	SUB 5	SUB 6
Relaxation	0.1585	0.0499	0.1863	0.2217	0.0556	0.1702
Standard time 70%	0.5746	0.1356	0.5628	0.3339	0.4569	0.802
Standard time 100%	0.4879	0.0862	0.2221	0.3027	0.3191	0.604

**Table 5:** RG Differences between relaxation state and product assembly at 70 and 100% ST for all subjects.

People	Relaxation versus 100% ST *p* value	Relaxation versus 70% ST *p* value
Person 1	*p* ≤ 0.001	*p* ≤ 0.001
Person 2	*p* ≤ 0.001	*p* ≤ 0.001
Person 3	0.443	*p* ≤ 0.001
Person 4	0.002	*p* ≤ 0.001
Person 5	*p* ≤ 0.001	*p* ≤ 0.001
Person 6	*p* ≤ 0.001	*p* ≤ 0.001

## Data Availability

The datasets generated during the current study are available from the corresponding author upon reasonable request.
